# Challenges and Complications in the Management of Advanced Oropharyngeal Carcinoma: Role of Post-Mortem Diagnosis and Future Perspectives

**DOI:** 10.3390/jcm13175198

**Published:** 2024-09-02

**Authors:** Francesca Consalvo, Matteo De Simone, Alfonso Scarpa, Alfonso Acerra, Francesco Antonio Salzano, Vittorio Fineschi, Alessandro Santurro

**Affiliations:** 1 Department of Medicine, Surgery and Dentistry, University of Salerno, 84081 Salerno, Italy; fconsalvo@unisa.it (F.C.); alfonso.acerra@unisa.it (A.A.); frsalzano@unisa.it (F.A.S.); asanturro@unisa.it (A.S.); 2 BrainLab s.r.l., Mercato San Severino, 84085 Salerno, Italy; 3 Unit of Legal Medicine, University Hospital “San Giovanni di Dio e Ruggi D’Aragona”, 84081 Salerno, Italy; 4 Unit of Otolaryngology, University Hospital “San Giovanni di Dio e Ruggi, D’Aragona”, 84081 Salerno, Italy; 5 Department of Anatomical, Histological, Forensic and Orthopaedic Sciences, Sapienza University of Rome, 00161 Rome, Italy; vittorio.fineschi@uniroma1.it

**Keywords:** head and neck, oropharyngeal carcinoma, squamous-cell cancer, lingual artery bleeding, autopsy investigation, post-mortem diagnosis

## Abstract

Oropharyngeal squamous-cell carcinoma (OPSCC) poses significant challenges in diagnosis, treatment, and management and has important medico-legal and forensic implications. In particular, the management of OPSCC and its treatment-related complications can often be challenging. In cases with advanced OPSCC, a loco-regional extension of the tumor can contribute to the destruction of oral cavity tissues, while the radiotherapy treatment can induce profound changes in tissue morphology and structure. These changes, which resemble tumor neoplasms and endovascular effects, are related to a higher risk of fatal bleeding, as reported in the case study illustrated, in which a hemorrhage occurred from a lingual artery, originating from an ulcerative, necrotic, hemorrhagic lesion on the tongue. Bleeding complications in OPSCC and prolonged radiotherapy are associated with high mortality and require comprehensive management strategies to improve survival and quality of life. Autopsy investigations, contributing to the definition of post-mortem diagnosis, can provide valuable insights into the pathogenetic mechanisms underlying bleeding and guide therapeutic decisions and preventive measures. The integration of autopsy and histopathological investigation into clinical practice should be considered as a necessary support to optimize the management of complications in advanced OPSCC patients, emphasizing the importance of a patient-centered approach and continued research.

## 1. Introduction

Head and neck squamous-cell carcinoma (HNSCC), which includes cancers of the oral cavity and oropharynx, is the world’s sixth most common cancer [1].

Oropharyngeal squamous-cell carcinoma (OPSCC) arises from various sites within the oropharynx, including the soft palate, tonsils, base of the tongue, pharyngeal wall, and vallecula. The most common anatomic locations for OPSCCs are the tonsillar complex and the base of the tongue, which account for 96% of oropharyngeal tumors [2].

Traditionally associated with alcohol and tobacco use, the epidemiology of OPSCC has undergone a notable shift due to declining smoking rates and the emergence of human papillomavirus (HPV) as a significant etiological factor [3,4,5]. The incidence of HPV-related OPSCC has risen sharply in high-income countries, with an estimated 55,000 cases and 12,000 deaths annually in the United States alone [6].

The advent of prophylactic HPV vaccination holds the promise of preventing a substantial proportion of OPSCC cases, with HPV-16 implicated in most infections [7,8]. The American Joint Committee on Cancer’s (AJCC) distinction between HPV-positive (HPV+) and HPV-negative (HPV-) OPSCC underscores the different molecular profiles, tumor characteristics, and treatment outcomes [9,10].

The clinical presentation of OPSCC includes a variety of symptoms such as pharyngeal swelling, dysphagia, visible mass, and otalgia, which often mimic benign conditions and require careful diagnostic evaluation [11]. Histologically, OPSCC has several subtypes, each of which has prognostic implications. Prognostic indicators for local recurrence and overall survival include tumor diameter, nodal status, surgical margins, degree of differentiation, invasion pattern, and patient response [12,13].

The management of OPSCC is a comprehensive strategy that encompasses various modalities, including surgery, radiotherapy, chemotherapy, or a combination of these, tailored to the specific type of cancer, its location, and the stage of the disease. The selection of treatment modalities is a careful consideration based on the individual characteristics of the patient’s cancer. Despite advancements in treatment, the nature of OPSCC therapy introduces the potential for long-term complications that significantly impact survivorship and quality of life [14,15]. As the field of oral cancer management evolves, strategies must continually adapt to address and mitigate the potential complications of OPSCC treatment. Achieving a balance between the efficacy of treatment and the possible complications is a complex challenge, necessitating ongoing research and a patient-centered approach to care [16,17,18,19].

In the OPSCC treatment and complications scenario, toxicities are known to be strongly related to the dose of radiotherapy received by normal tissues. Severe late toxicities have been reported in 43% of patients after radiotherapy treatment, and toxicity of this magnitude can last a lifetime; in light of this, it is imperative to implement dose tailoring strategies that are as short as possible to limit the damage. In this direction, several de-escalation studies have been published, such as the PATHOS (Postoperative Adjuvant Treatment for Human Papillomavirus (HPV)-positive Tumors) study, which aims to demonstrate that de-escalation of adjuvant treatment, stratified by risk, not only maintains high survival and short recurrence rates in patients with HPV-positive OPSCC but also improves long-term swallowing function. In any case, the rationale of all these studies is that we need to balance and mitigate the subject’s level of risk of recurrence, keeping in mind that 50–60 Gy is a critical dose range with regard to the risk of long-term dysphagia and radiotherapy-related dystrophy bleeding [20].

In addition to these clinical complexities of managing advanced OPSCC, it is critical to consider the medico-legal and forensic implications that may arise in such cases. The intersection of medical care, legal considerations, and forensic analysis plays a critical role in understanding the circumstances surrounding patient care, treatment decisions, and adverse outcomes in cases of advanced oropharyngeal carcinoma [21,22,23].

## 2. Clinical Framing and Treatment

The clinical presentation, prognosis, and treatment of oropharyngeal squamous-cell carcinoma (OPSCC) are influenced by several factors, including sex, age at diagnosis, and cancer stage.

Sex differences in OPSCC presentation and prognosis have been reported, with men generally exhibiting a higher incidence of OPSCC attributed to a higher prevalence of risk factors such as tobacco and alcohol use [24,25]. Additionally, human papillomavirus (HPV) infection is more common in men, contributing to differences in tumor biology and clinical outcomes [26].

Sex may influence treatment selection and response in OPSCC. Adjuvant therapies such as chemotherapy and radiotherapy play an important role in the treatment of OPSCC. However, the response to adjuvant therapies may vary according to sex. For example, women have been shown to have a higher susceptibility to certain treatment-related toxicities such as mucositis and dermatitis [27]. These differences in treatment tolerability and toxicity profiles must be carefully considered when tailoring adjuvant therapies for OPSCC patients based on their sex. Strategies such as dose adjustments or alternative treatment options can be used to minimize adverse effects and optimize treatment outcomes [28,29].

Age is another important factor to consider in the treatment of OPSCC because the epidemiology of the disease and tumor biology differ across age groups. OPSCC is typically diagnosed in two different age groups: younger patients (typically under 45 years of age) and older patients (over 55 years of age) [30]. Younger patients are more likely to have HPV-associated OPSCC, which tends to have a more favorable prognosis [31]. In contrast, older patients are more likely to have HPV-negative tumors and may have more frequent comorbidities [32,33,34].

Accurate staging of OPSCC is paramount for treatment planning and prognosis prediction. Staging systems (SS) like the American Joint Committee on Cancer (AJCC) SS provide standardized frameworks for classifying OPSCC based on tumor size, lymph node involvement, and distant metastases, aiding in treatment decisions and prognostic assessments [35]. Early-stage OPSCC, typically classified as T1-T2 tumors, can often be effectively treated with single modalities like surgery or radiation therapy. Minimally invasive surgical techniques such as transoral robotic surgery (TORS) and transoral laser microsurgery (TLM) offer improved functional outcomes and reduced morbidity compared to traditional open surgery [36,37]. Conversely, advanced-stage OPSCC may necessitate multimodality treatment approaches involving surgery, radiotherapy, and chemotherapy [38].

Despite optimal initial treatment, some OPSCC patients face disease recurrence or persistence, leading to the consideration of salvage therapies such as re-irradiation or systemic therapy based on disease extent and patient health status [39,40,41]. Recent research has focused on de-escalating treatment intensity to minimize long-term toxicities while maintaining disease control, emphasizing personalized treatment approaches tailored to individual patient factors and tumor characteristics [42,43,44].

The management of OPSCC is a multifaceted journey that, while striving for optimal therapeutic outcomes, is not without potential complications linked to both the disease itself and the modalities employed in its treatment.

Surgery, particularly transoral procedures like transoral robotic surgery (TORS) and transoral laser microsurgery (TLM), is crucial for managing early-stage OPSCC and when organ preservation is not feasible.

## 3. Complications

Complications such as postoperative hemorrhage, infection, and functional impairments can arise [45,46,47]. Neck dissection, a common procedure, may lead to shoulder dysfunction and lymphedema [48,49]. Radiation therapy, often combined with chemotherapy, is a primary treatment for OPSCC, causing acute complications like mucositis and xerostomia [50,51,52,53]. Late complications include radiation-induced fibrosis and osteoradionecrosis [54,55]. Chemotherapy, especially platinum-based agents, can result in hematological toxicity and gastrointestinal symptoms [56,57,58]. Targeted therapies like EGFR inhibitors and immune checkpoint inhibitors have shown promise but may lead to skin rash and immune-related complications, respectively [59,60,61,62].

Combined treatment with chemotherapy and radiotherapy can lead to various side effects, encompassing skin and mucosal erosion, xerostomia, underlying soft tissue fibrosis, and osteoradionecrosis. Further complications include secondary effects on the surrounding vasculature, including premature atherosclerosis with stenosis and arterial wall weakening due to adventitial fibrosis, elastic filament fragmentation, and vasa vasorum destruction [63].

Among the complications of treatment for OPSCC, oropharyngeal hemorrhage is a rare but life-threatening occurrence [64,65]. It may occur spontaneously after radiochemotherapy due to arterial wall weakening, with vascular erosion more common in advanced or recurrent tumors, infections, and pharyngocutaneous fistulas [66]. The involvement of external carotid artery branches, especially in locally advanced cases, heightens the bleeding risk.

Particularly concerning are cases of hemorrhage in patients without tumor recurrence, where fatality rates can reach 40%. The facial artery and lingual artery are primary sources of such hemorrhage [67].

Oropharyngeal hemorrhage presentations vary based on bleeding severity, encompassing hemoptysis, hematemesis, epistaxis, and persistent or acute hemorrhages. Bleeding may obstruct airways, lead to blood aspiration, and result in asphyxiation. In most cases reported in the literature, it is noted that a major hemorrhagic event is preceded by minor ones, which can serve as a warning sign to identify at-risk patients [68,69].

Operations for oropharyngeal hemorrhage are intricate due to radiation-induced damage, hemodynamic imbalance, and difficulty in localizing the hemorrhage. Endovascular management has demonstrated superior safety and efficacy compared to surgical approaches, effectively halting oropharyngeal hemorrhage [70,71]. For patients with oropharyngeal neoplasms treated with RT who show recurrent bleeding episodes and mucosal ulceration, particularly after the acute treatment phase, hospitalization with prophylactic surgical ligation or embolization of the affected arteries may be recommended [72,73,74].

## 4. Role of Post-Mortem Investigations and Diagnosis

Post-mortem investigations can often play a key role in evaluating the findings and defining the cause of death in cancer patients and fully understanding the complications, providing a comprehensive analysis of the patient and enabling targeted sampling that would otherwise not be possible [75,76].

Autopsy investigation in cases with a clinical history of advanced neck-oropharyngeal carcinoma should always be conducted following a rigorous autopsy protocol aimed at identifying and documenting the characteristics and extent of the oropharyngeal carcinoma, as well as any elements that might help in determining the cause of death [77]. In fact, a fundamental objective of the autopsy in these cases is represented by the correct identification, documentation, and interpretation of the pathological findings.

On the other hand, it is important to take into consideration that the neck poses significant challenges to the forensic pathologist for several reasons. First of all, the anatomical localization of the alterations, often located in regions of the neck that, due to the conformation and anatomical relationships of the cervical organs, do not allow optimal visualization of the possible findings, often not even with particularly invasive anatomical dissections. In these cases, in fact, the anterior neck structures can also present anatomical variations and characteristics that could make it difficult to distinguish artifacts from pathological findings. Secondly, pathological features may have a degree of expressiveness that complicates the interpretation of post-mortem findings in the neck.

To reduce the practical difficulties in interpreting autopsy results in the neck, important factors must be considered. First, it is essential that the neck is properly dissected during the post-mortem investigation by autopsy. This involves a layer-by-layer dissection of the neck after vascular decompression of the neck by removal of the brain and viscera. Dissection of the neck is best achieved by a series of incisions that maximize exposure of the ventral, lateral, and submental portions of the neck. This dissection may be extended to include the face. Secondly, it is important to be proactive in recognizing the pitfalls and artifacts that may become apparent based on the history, site, and circumstances of the case, always taking into consideration the possible diagnostic traps related to the misinterpretation [78].

The forensic approach first should include a detailed review of the pre-mortem medical records, with particular attention to the patient’s oncological history and treatments received. During the external examination, external signs should be identified and documented, and the neck area should be carefully examined.

As an adjunct and preliminarily to autopsy, post-mortem TC (PMCT) could be useful in selected cases since post-mortem imaging can aid the forensic pathologist, helping in planning the dissection, visualizing any alterations, and helping to clarify the location and extent, as well as the type of any pathological alterations [79,80,81,82,83].

Dissection of the neck structures and internal examination should be performed starting with a U-shaped skin incision on the anterior surface of the neck and thorax (mastoid process → postero-lateral surface of the neck → outer and middle third of the clavicle → sternal angle, and the same on the other side up to the mastoid). This approach allows wide access to the area under investigation and complete visualization of the cervical structures. A progressive layer-by-layer dissection of the structures should be performed to investigate the different anatomic regions and zones of the neck. This dissection, in fact, provides for maximal exposure of the ventral, lateral, and submental portions of the neck. After isolating muscle layers one by one and exposing the cervical organs, an en-bloc dissection of the oro-cervico-respiratory block (tongue–hypopharynx–larynx–trachea–bronchi–lungs), according to the Ghon technique, allows a better visualization of the entire anatomic region, preserving the integrity and anatomic rapports between the cervico-oropharyngeal structures.

Following the described approach, oropharyngeal alterations in head and neck cancer patients can be detected, documented, and characterized, with the aim of understanding the pathophysiological mechanisms related to death and giving a possible focus on the related risks, including in those patients seemingly under control.

Histopathological examination is crucial in these cases to document possible modification related to primitive cancer alterations and document the effects of radiotherapy in the head and neck region associated with severe morpho-structural and vascular alterations, including endovascular phenomena leading to possible fatal bleeding events.

In fact, from a histological point of view, tumor-like lesions manifest as extensive morpho-structural alterations, resembling tumor neoplasms, characterized by tissue necrosis, chronic inflammation, fibrosis, and vasculopathy [84,85]. Endovascular effects, on the other hand, involve pathological changes in blood vessels, such as vascular inflammation, fibrinoid degeneration, and vascular stenosis [86].

Overall, both tumor-like lesions and endovascular effects reflect the severe alterations induced by radiotherapy on the oropharyngeal tissue, predisposing patients to serious complications, including bleeding and further tissue damage. The importance of assessing the risk of fatal bleeding events in patients with advanced oropharyngeal carcinoma treated with radiotherapy, especially those with significant side effects, is therefore crucial.

## 5. Illustrative Case

As an illustrative case of post-mortem investigations and comprehensive analysis of OPSCC fatal complications, the case of a 69-year-old woman with a history of advanced oropharyngeal cancer suddenly died after experiencing a severe coughing fit with initial hemoptysis is reported.

Medical history indicated that the patient had initially sought medical attention for an ulcerating neoformation in the oral cavity. Subsequent examinations revealed a moderately differentiated squamous-cell carcinoma. Further investigations, including CT and PET scans, revealed extensive tissue involvement in the cervicofacial region and multiple lymphadenopathies. Diagnosed with cT4bN2c St. IVB, the patient underwent a tracheotomy and PEG placement due to upper airway issues and began treatment with chemotherapy and concomitant radiochemotherapy. Chemotherapy treatment was administered with Cisplatin, given on a weekly schedule for seven weeks at a dosage of 40 mg/m^2^. Radiotherapy was planned using intensity-modulated radiation therapy (IMRT) techniques. Iso-dose lines were drawn to ensure that the tumor areas received a total dose of 70 Gy, while the vulnerable structures surrounding the tumor area were protected with lower doses, not exceeding 54 Gy.

After treatment, positive results were seen with no loco-regional recurrence, with a six-month therapy-free interval. During this interval, the patient was regularly monitored with otolaryngological clinical examinations, initially monthly and then every two months. One year after tumor diagnosis, the oncological staging revealed advanced-stage disease with suspected lymph node and lung metastases (cT4a, cN2c, pN+, cM1 lymph nodes and lung, Stage IV C), leading to the initiation of biological therapy with Nivolumab, resulting in subsequent good control of neoplastic progression.

In this state of apparently good control of the neoplastic pathology, approximately seven months after the end of radiotherapy and concomitant chemotherapy, the patient died suddenly due to unexpected and unpredictable profuse oropharyngeal bleeding. There were no previous episodes of minor bleeding to suggest sudden fatal major bleeding.

An autopsy was performed to determine the origin of the bleeding, to define any potential triggering and causative factors that led to the unforeseen and tragic event, and so to establish the cause of the death.

### 5.1. Autopsy Findings

The external examination was unremarkable except for widespread staining of the clothing with blood.

At autopsy, the oral cavity was filled with blood. The tongue showed an ulcerous, necrotic, hemorrhagic lesion extending from the lateral margin of the right portion of the tongue, penetrating the muscular body of the tongue and involving its entire thickness. 

The lungs showed a pale appearance in the mid-apical region and a hematogenous distribution in the lower lobes. The airways contained endoluminal blood material from inhalation. The gastrointestinal tract and stomach, up to the pylorus, contained a significant amount of brownish fluid material consistent with ingested blood.

**Figure 1:**
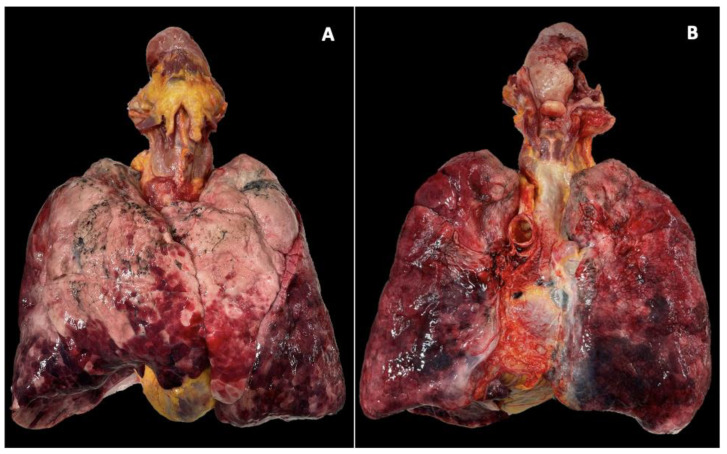
Oro-cervico-respiratory block according to the Ghon technique (en bloc). There is a noticeable pale appearance in the mid-apical region of the lungs, and blood is spreading at the bases. The tongue showed an ulcerous, necrotic, hemorrhagic lesion extending from the lateral margin of the right portion of the tongue, penetrating the muscular body of the tongue and involving its entire thickness.

**Figure 2:**
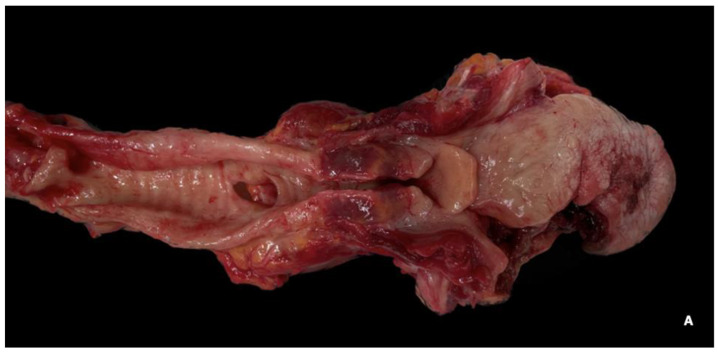

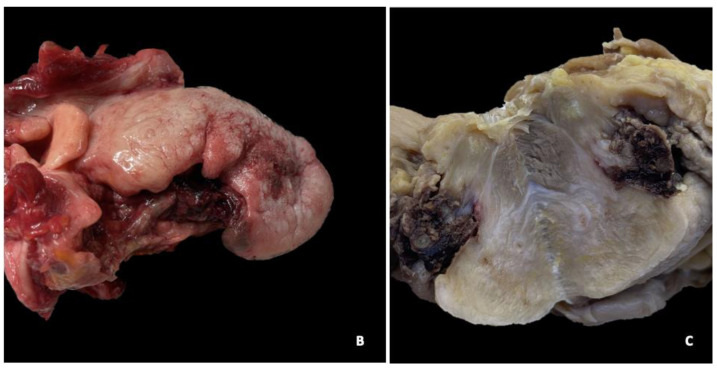
(**A**,**B**) The tongue exhibited an ulcerous, necrotic, hemorrhagic lesion that extended from the lateral margin of the right portion of tongue, penetrating the muscular body of the tongue and affecting it throughout its thickness. (**C**) Close, serial sections of the ulcerative lesion after formalin fissation revealed necrotic tissue extended and dissociated from hemorrhagic phenomena. Within the necrotic tissue, a vascular branch (branch of the lingual artery) was identified as possible source of the bleeding.

The oro-cervico-respiratory block (tongue–hypopharynx–larynx–trachea–bronchi–lungs) was removed in its entirety, according to the Ghon technique (*en bloc*), and the tongue–hypopharynx–larynx–trachea portion was examined after formalin fixation to enhance the study of anatomical structures of interest. (Figure 1) The tongue showed a deep ulceration of approximately 2 × 2 cm in the lateral margin of the right portion of the tongue, extending 13 mm into the muscular body of the tongue, affecting the organ through its entire thickness on both the upper and lower surfaces, and reaching approximately 7 mm from the lingual frenulum. (Figure 2A–C)

Close, serial sections of the ulcerative lesion revealed necrotic tissue extended and dissociated from hemorrhagic phenomena. Within the necrotic tissue, a vascular branch (branch of the lingual artery) was identified as the source of the bleeding.

### 5.2. Histopathological Examination

**Figure 3:**
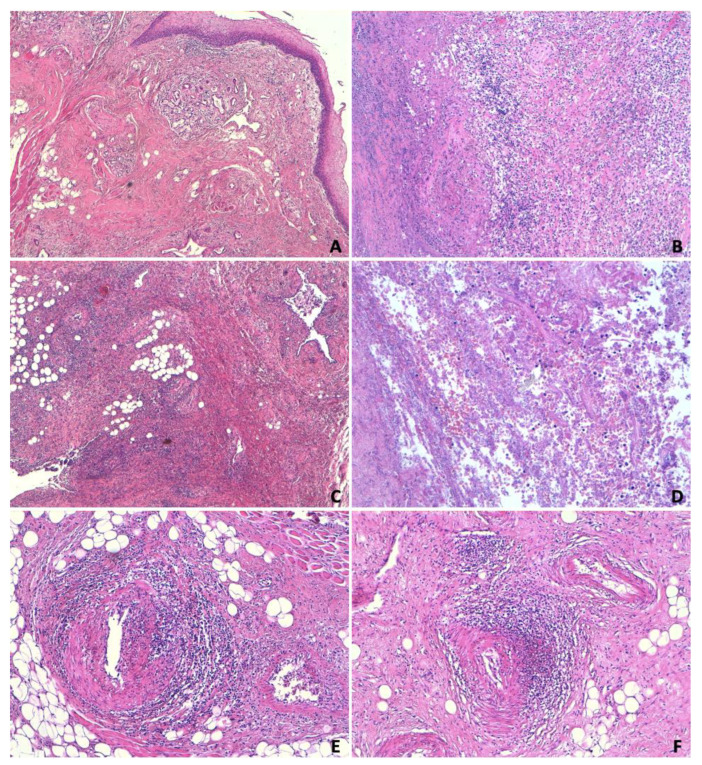
Extensive necrotic, hemorrhagic ulceration of the tongue involves both the lateral basal plane and the superficial squamous epithelial lining. (**A**) The base and edges of this ulceration are covered by a thick layer of necrotic, hemorrhagic material containing assessable cellular debris. (**B**) The underlying muscle-connective tissue layers host extensive necrotic, granulocytic phenomena (presumably secondary to bacterial superinfection), within which decaying food residues, microbial colonies, and various nerve trunk structures infiltrated by inflammatory elements are identified. (**C**,**D**) In the sublingual areas corresponding to the aforementioned ulceration, islands of glandular tissue (attributable to minor salivary glands) with severe dysplastic morpho-structural alterations are present, sometimes giving the picture of a “tumor-like lesion” correlated with the cytolysis effects of prolonged radiotherapy. (**E**) Adjacent tissues show arteriolar vascular branches with endovascular phenomena causing subtotal luminal stenosis and perivascular changes. (**F**) The lingual body shows lympho-granulocytic inflammatory infiltrates and focal necrotic, hemorrhagic phenomena, resulting in the fragmentation of striated muscle fibers.

All tissue samples were fixed in formalin, embedded in paraffin, and subjected to routine microscopic histopathological examination using hematoxylin and eosin (H&E) staining. (Figure 3)

Microscopic examination of formalin-fixed paraffin-embedded tissue sections stained with H&E confirmed the necrotic, hemorrhagic ulceration of the tongue. In the sublingual areas corresponding to the ulceration, islands of tissue with severe morpho-structural alterations, sometimes resembling a “tumor-like lesion”, were present. Adjacent tissue areas showed arteriolar vascular branches with endovasculitic phenomena. The muscular body of the tongue showed lympho-granulocytic inflammatory infiltrates and focal necrotic, hemorrhagic phenomena, resulting in the fragmentation of the muscle-striated fibers. These alterations were attributed to the cytolytic effects of prolonged radiotherapy. On the other hand, the original neoplastic pathology could not be detected.

Brain samples displayed both intracellular and extracellular cerebral edema. The lungs showed alveolar spaces containing erythrocytes. No further significant findings were found in the remaining tissue samples.

In view of these findings, the cause of death was identified as hemorrhage from an ulcerated, necrotic, hemorrhagic lesion on the tongue in a patient with advanced oropharyngeal carcinoma. The loco-regional extension of the tumor contributed to the destruction of oral cavity tissues, while the radiotherapy treatment induced morpho-structural changes, predisposing the patient to bleeding unexpectedly without premonitory signs, suggesting the need for preventive treatment.

In fact, investigations revealed significant alterations attributable to the cytolytic effects of prolonged radiotherapy. Histopathological examination showed radiotherapy-related severe morpho-structural and vascular alterations, including endovascular phenomena, leading to fatal bleeding.

On the other hand, the reported illustrative case provides food for thought regarding the management of OPSCC and treatment-related complications and the importance of post-mortem investigations in understanding these adverse fatal events, highlighting the need for complete and meticulous examination and collaboration between multidisciplinary experts.

## 6. Future Perspectives

The current literature only partially addresses OPSCC complications. Bleeding complications in OPSCC and prolonged radiotherapy are associated with high mortality and require comprehensive management strategies to improve survival and quality of life.

This study offers a proof of concept that may improve the diagnostic possibilities and the ability to detect the anomalies developed in this type of patient. Autopsy investigations, contributing to the definition of post-mortem diagnosis, can provide valuable insights into the pathogenetic mechanisms underlying bleeding and guide therapeutic decisions and preventive measures. The integration of autopsy and histopathological investigation into clinical practice should be considered as a necessary support to optimize the management of complications in advanced OPSCC patients.

Subsequently, based on this evidence, treatment strategies can be developed in the future, strengthened by the ability to identify early on which of these patients are most prone to these complications. As an example, a personalized approach, also with an artificial intelligence-assisted decision and operative support, may help in better planning of treatment both in terms of doses and in terms of targeting, fully integrating the so-called adaptive radiotherapy. In parallel, the most important steps in radiotherapy preparation, delivery, and evaluation, as well as radiotherapy planning, can consequently be improved by also considering the different complications detected through these multidisciplinary approaches.

## 7. Conclusions

Bleeding in OPSCC is a dreaded complication associated with high mortality rates and a poor short- and mid-term prognosis. The management of complications associated with OPSCC and its treatments is crucial to improving survival and quality of life. The delicate balance between treatment efficacy and complications requires continuous research and a patient-centered approach to care.

Post-mortem investigations can often play a key role in giving valuable insights into understanding and managing this serious complication. In this scenario, post-mortem findings can provide a solid scientific basis for elucidating the pathogenetic mechanisms underlying bleeding and assessing the impact of radiation therapy on tissue structure and function.

Furthermore, the importance of rigorous postoperative monitoring is evident in order to mitigate risks associated with surgical and combined radiotherapy-chemotherapy treatments. Clinicians should prioritize strategies that reduce the incidence of severe complications, and emerging protocols should incorporate risk management for spontaneous hemorrhage, particularly in patients with advanced or recurrent disease.

The implications for future guidelines are significant: recommendations for dose tailoring, long-term patient monitoring, and interdisciplinary approaches are important to manage the complex needs of OPSCC patients. Additionally, given the possible severe risks associated with combined treatments, there is a critical need for further research into optimizing therapeutic combinations to balance efficacy with patient safety [87].

## Data Availability

The data are included in a legal case in Italy, and for this reason, they are not available.
